# Alterations of gut viral signals in atrial fibrillation: complex linkage with gut bacteriome

**DOI:** 10.18632/aging.204222

**Published:** 2022-08-18

**Authors:** Kun Zuo, Jing Li, Chen Fang, Jiuchang Zhong, Li Xu, Xinchun Yang

**Affiliations:** 1 Heart Center and Beijing Key Laboratory of Hypertension, Beijing Chaoyang Hospital, Capital Medical University, Beijing 100020, China

**Keywords:** atrial fibrillation, gut viral signals, metagenome, virus-bacteria linkages, predictive model

## Abstract

The gut microbiota has a known complex association with atrial fibrillation (AF) progression, but the association of gut viruses with AF is undefined. Metagenomic data in a cohort of 50 AF patients and 50 matched controls were examined to profile the gut viral signals and determine their associations with intestinal bacteria and the AF phenotype. The gut viral alterations were examined, and the marked elevation of viral diversity, including increased Simpson, Shannon, and Pielou index, was revealed in AF patients. The specific alteration of the intestinal viral population, such as overgrowth of *Streptococcus virus DT1* and *Pseudomonas phage*, as well as imbalanced gut viral function, dominated by integral component of the membrane, and metal ion binding were detected in AF patients. Moreover, regarding co-occurrence networks connecting viruses and bacterial organisms, increasingly disordered virus-bacteria linkages were seen in AF cases with severe AF progression. Notably, the associations of *Synechococcus phage S−SM1* and *Cronobacter phage CR5* with bacterial species were very tight in control individuals but markedly dampened in AF cases. Furthermore, the viral score built by the selected discriminative taxa between AF cases with or without recurrence after ablation was still significantly associated with recurrence (HR = 2.959, *P* = 0.0085), with a survival AUC of 0.878. We demonstrated for the first time that gut viral signatures are associated with AF, and suppressed viral-bacterial associations in AF suggest the gut virus might participate in AF progression, which has a potential value in predicting ablation outcomes.

## INTRODUCTION

Atrial fibrillation (AF), one of the most common arrhythmias health and socioeconomic burdens, has complex pathophysiology and significantly contributes to enhanced morbidity and mortality [1]. Recent evidence suggests a regulatory role for the gut bacteria in human health, including the influence on host metabolism and immune homeostasis in several cardiovascular diseases [2] such as hypertension [3], heart failure [4], atherosclerosis [5], and AF [6–8]. Notably, many AF patients (approximately 40%) suffered from gastrointestinal comorbidities, predominantly dyspepsia [9]. Therefore, the role of the gut environment or microbiome deserves more attention in the context of AF pathogenesis.

However, researchers are still striving to determine what shapes the microbial community. Some changes are driven by the powerful external influence of the environment and lifestyle [5]. However, the internal micro-environment of gut bacteria is the gut microbiome, a complex ecosystem that contains communities of bacteria, archaea, unicellular eukaryotes, multicellular eukaryotes, and viruses. Sharing the same host niches, these communities compete against, synergize with, and/or antagonize each other, potentially impacting the host [10].

With recent progress in high-throughput sequencing technology and data mining strategies, unculturable viral communities should not be underestimated as “dark matter”, whose number likely far surpasses those of bacterial populations [11–13]. The intestinal virome in healthy individuals includes eukaryote-infecting viruses and bacteriophages. Intestinal bacteriophages are substantially diverse among individuals and temporarily stable [11]. These viruses latently infect bacteria and co-evolve with intestinal bacteria, shaping bacterial ecology and acting as a driving factor in bacterial diversification and community composition through predation and horizontal gene transfer [10].

Currently, few reports have assessed the gut virome in AF patients. To examine the regulatory roles of intestinal viruses, it is urgent to determine their composition and functions. Hence, in this study, we performed analyses based on our previously published metagenomic dataset about AF, addressing the potential changes in virus composition and functions and the subsequent effects on clinical AF progression. More importantly, we proposed to reveal the importance of assessing the virus as an essential part of the microbiome ecological network based on the trans-kingdom interaction between virus and bacteria. In addition, this study evaluated the viral signal for its potential diagnostic potential in recurrent AF prediction after catheter ablation.

## RESULTS

### Data processing and taxonomic profiles of the gut viral signals in AF

Based on the metagenomic assembly, 857,736 contigs were obtained, with an average N50 length of 4,123 bp, ranging between 1,000 and 348,062 bp. Briefly, the comparison of bacterial and viral reads in non-AF controls and AF showed an elevation of relative proportions of the virome in the gut environment, with an average of 3,451,194.14 and 3,113,567.34 viral reads in AF cases and non-AF controls, respectively, and 49,676,139.2 and 44,817,408 bacterial-reads, respectively (Supplementary Figure 1A, 1B). Altogether, viral taxa, including 13 orders, 128 families, 756 genera, and 4,168 species, were annotated in the Kraken database, and relative abundance levels were calculated (Supplementary Figure 1C, 1D).

Virome differences could be driven by vertebrate viruses or bacteriophages. Next, the virome was examined at the order level for similarity to eukaryotic viruses or bacteriophages, based on the viral host information downloaded from the international committee on taxonomy of viruses (ICBV). Globally, 53.85% of all viral orders were known vertebrate-infecting viruses, and 15.38% were matched to bacteriophages’ reference genomes. Notably, AF was associated with a tendency towards the enrichment of phages (Supplementary Figure 1E). The above data indicated dysbiosis in bacteriophages is important in AF, with some vertebrate-infecting viruses showing an increased abundance in non-AF individuals, pointing to a potential linkage between bacterial dysbiosis and bacteriophage expansion in AF.

Further, we assessed whether these community bacteriophages were mostly lytic or temperate by detecting three markers of temperate phages in the (1) bacteria reference genome from the NCBI database, (2) prophage genes from the ACLAME database, and (3) Uniprot [14]. The abundances of lysogenic phages were calculated based on the species level, and we found that the proportion of lysogenic phages showed an increasing tendency throughout non-AF controls, PAF, and psAF (Supplementary Figure 1F). These findings might suggest that the intestinal virus mainly comprises temperate bacteriophages.

### Viral diversity in AF

**Figure 1:**
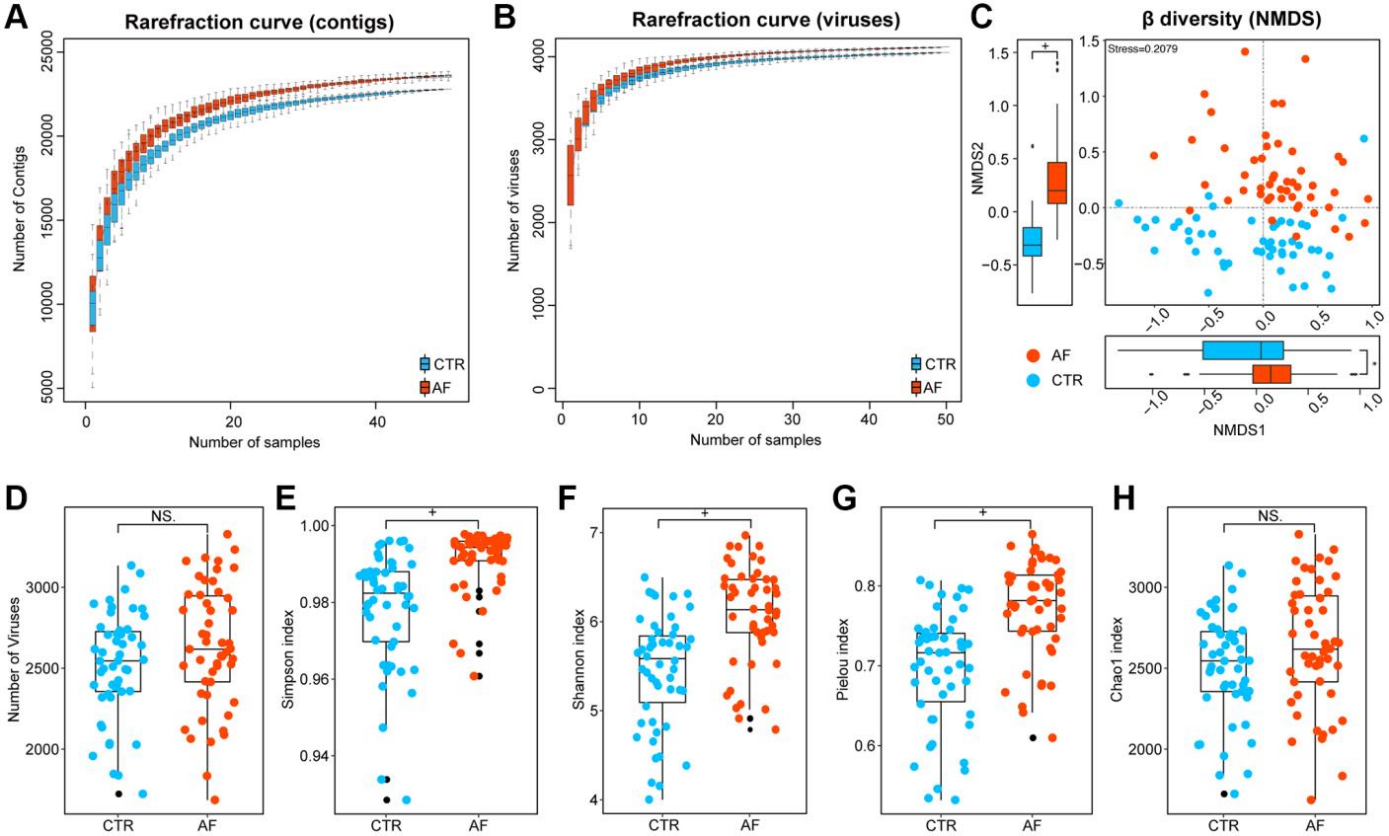
**Viral diversity in AF.** Rarefaction curves depicting viral contigs (**A**) and virus (**B**) numbers in control and AF specimens. Beta diversity by nonmetric dimensional scaling (NMDS) at the species level (**C**). Alpha diversity indexes, including the number of viruses (**D**), the Simpson index (**E**), the Shannon index (**F**), the Pielou index (**G**), and the Chao 1 index (**H**) at the species level in controls and AF cases. + denotes *p*-value < 0.01, ^*^p < 0.05, and NS indicates no significant difference based on Wilcoxon rank-sum test.

Rarefaction analysis was carried out by enumerating viral contigs (Figure 1A) and viruses (Figure 1B) in sample pairs, and the results showed that the accumulation curves for the totality of specimens nearly plateaued, suggesting most viral contigs and viruses could be detected. Specifically, the scatter plot of nonmetric dimensional scaling (NMDS) based on the abundances of viruses at the species level separated control and AF patients (*P* = 0.0403 for NMDS1; *P* = 1.25e−14 for NMDS2, Figure 1C), indicating different gut viral structures between the two groups. Moreover, α diversity (reflected by the Shannon index, Simpson index, and Pielou evenness at the species level) was markedly elevated in AF cases compared with control individuals (*P* = 0.0953 for the number of viruses, Figure 1D; *P* = 1.74e−08 for the Simpson index, Figure 1E; *P* = 7.05e−07 for the Shannon index, Figure 1F; *P* = 2.05e−07 for Pielou evenness, Figure 1G; *P* = 0.0953 for the Chao1 index, Figure 1H).

To address whether the observed alteration of viral signals was influenced by traditional AF risk factors, multivariate linear regression analysis for age, BMI, HTN, T2DM, TC, and medication was performed. Also, the independent strength of association between AF and the viral signatures of alpha diversity was examined. The results showed that AF was associated with increased viral Shannon index, Pielou evenness, and Simpson index independent of age, gender, BMI, TC, HTN, T2DM, or medication (Supplementary Table 1, Supplementary Figure 2). Therefore, it was concluded that the contribution of confounders to disordered viral signals was less than that of AF.

### Altered gut viral composition in AF

**Figure 2:**
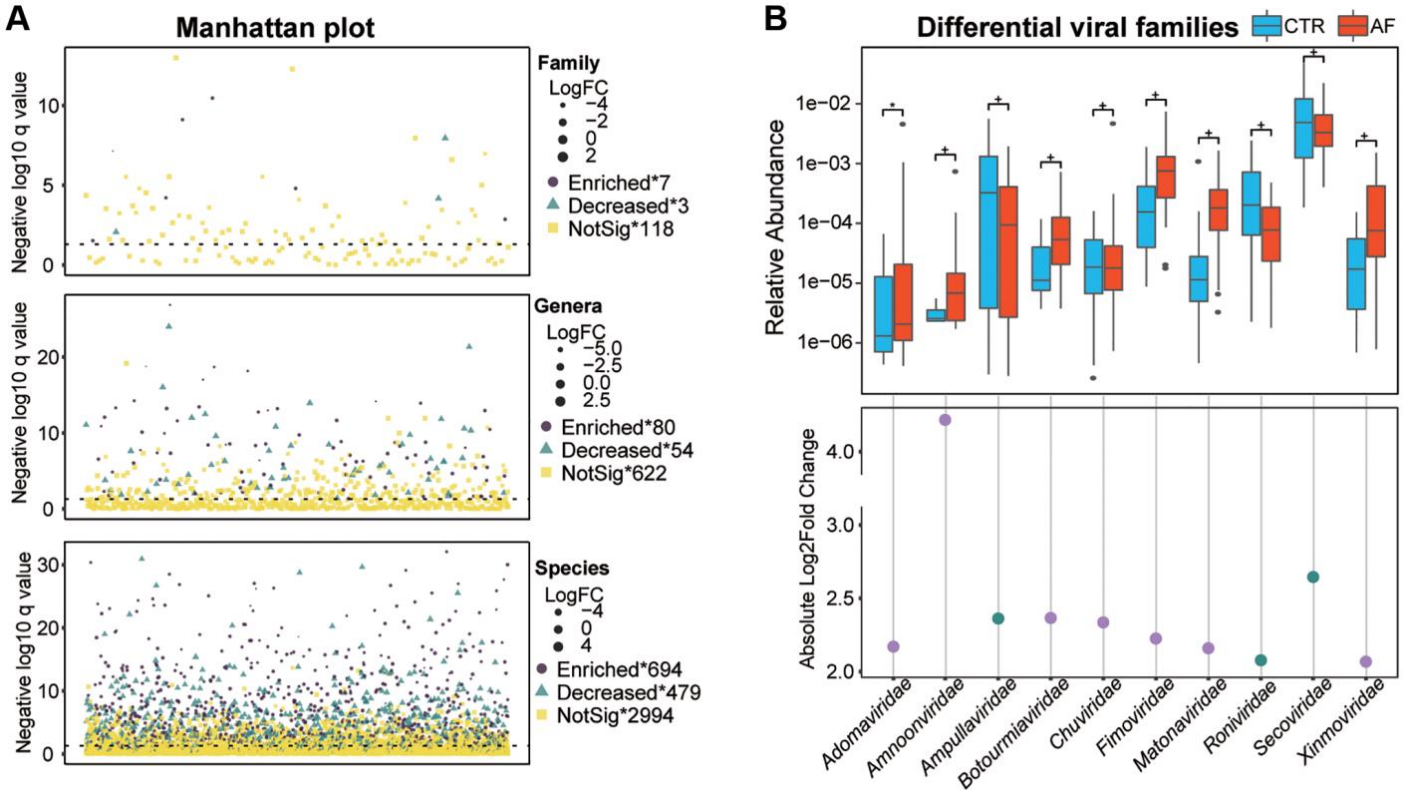
**Altered gut viral composition in AF.** The Manhattan plot shows an overview of differentially enriched viral taxa between non-AF and AF individuals (**A**). Box plots of differentially enriched viral families (**B**) between non-AF control and AF individuals. Boxes are interquartile ranges; lines denote medians; circles are outliers. The scatter plot shows absolute values of Log 2 (fold change of CTR/AF), while dots colored in purple and green denote enriched viruses in AF and controls, respectively. + denotes adjusted *p* (*q*) value < 0.01, ^*^q < 0.05, and NS indicates no significant difference based on DESseq analysis.

DESeq analysis was performed to identify taxa differentially with the standard of adjusted *p* < 0.05 and |Log2 (fold change) |>2 between controls and AF at the family, genus, and species levels. A total of 10 families, 134 genera, and 1,173 species were identified as differential taxa between non-AF controls and AF cases, and more taxa, including 7 families, 80 genera, and 694 species, were found to be significantly enriched in AF cases than in control patients (Figure 2A, Supplementary Table 2). There was a higher abundance of families such as *Secoviridae* and *Fimoviridae* in the gut of AF patients (Figure 2B).

### Functional changes in the intestinal viral signals in AF

**Figure 3:**
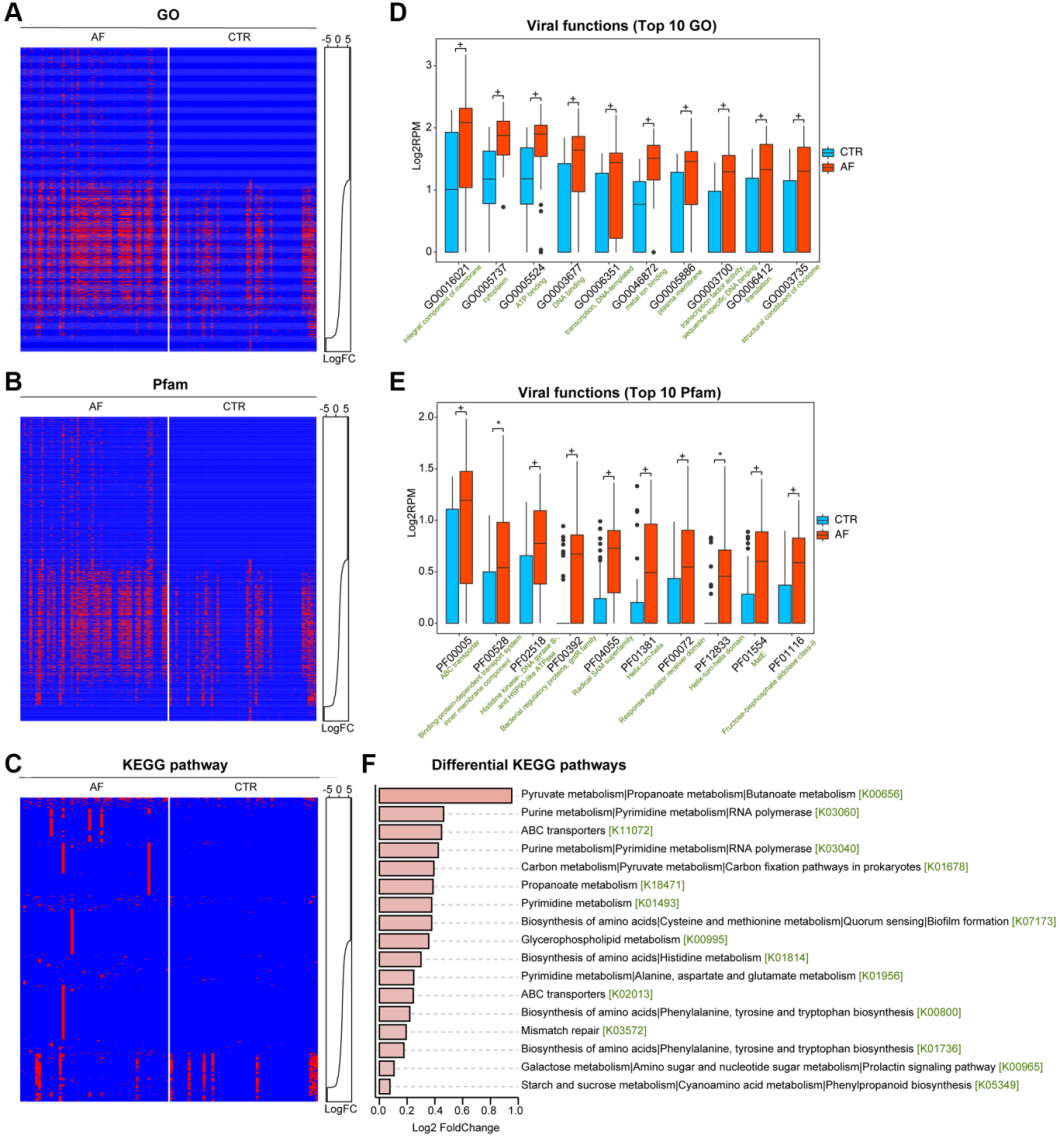
**Functional alterations of the gut viral signals in AF.** Presence-absence heat map of categorized viral functions in non-AF and AF individuals, including in Gene Ontology (GO, **A**), the Pfam protein family database (**B**), and the KEGG pathway (**C**). The abundance distributions were graphed with line charts, and abundance levels were presented as Log2 reads per million mapped reads (RPM). The box plots show the top 10 differential viral functions (**D** for GO and **E** for Pfam). Boxes represent interquartile ranges; lines denote medians, and circles are outliers. + denotes adjusted *p* (*q*) value < 0.01, ^*^q < 0.05, and NS indicates no significant difference based on limma analysis. (**F**) Differential abundance in the discriminative KEGG pathways in AF compared to the control group.

To examine gut viral functions, a HUMANN2-based assessment of reads of viral contigs in the GO, Pfam protein family, and the Kyoto Encyclopedia of Genes and Genomes (KEGG) databases was conducted. We compared the predicted gut viral functions between non-AF controls and AF cases. Interestingly, AF patients had altered viral functions, with 505 GO (Figure 3A), 518 Pfam protein functions (Figure 3B), and 17 KEGG pathways (Figure 3C; Supplementary Table 3), where *P* < 0.05 was considered statistically significant based on limma analysis. Of these, GO terms such as integral component of the membrane, cytoplasm, ATP-binding, DNA binding, and metal ion binding (Figure 3D), Pfam proteins such as ABC transporter, Histidine kinase-, and DNA gyrase B- (Figure 3E), as well as KEGG pathways such as Glycerophospholipid metabolism, Purine metabolism, Pyrimidine metabolism, RNA polymerase (Figure 3F), were the prominent proteins/functions, indicating the essential roles of viruses in virus-host interactions and the potential interconnection of gut viruses and bacterial organisms.

### Virus-bacteria co-occurrence networks

**Figure 4:**
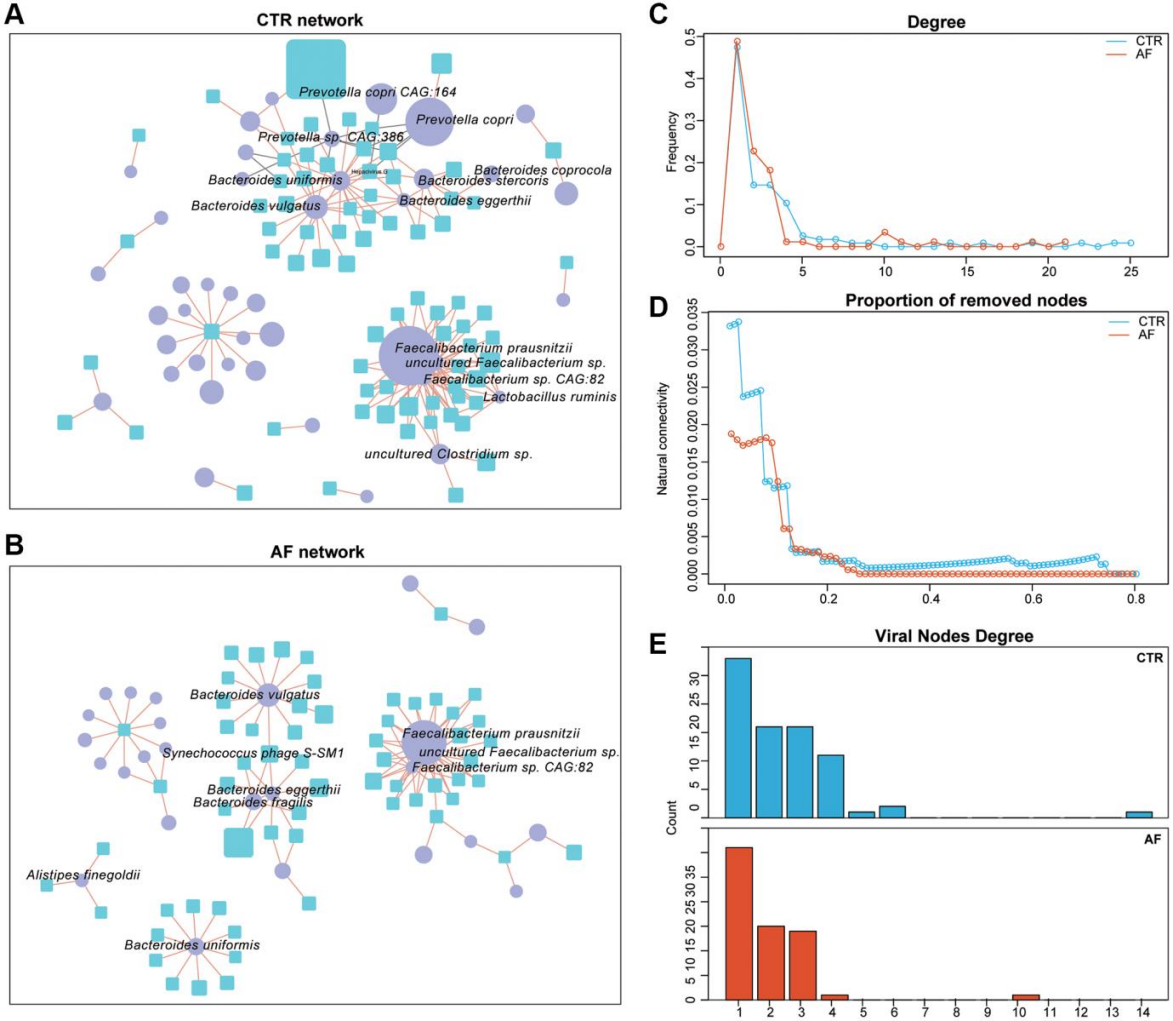
**Virus-bacteria co-occurrence networks.** Virus-bacteria co-occurrence networks for non-AF controls (**A**) and AF patients (**B**). The nodes are viruses and bacterial species. Purple ellipses and blue rectangles represent bacterial species and viruses, respectively. Edges represent positive (orange) and negative (grey) associations of viruses with bacterial organisms at the species level. Nodes denote distinct bacterial organisms or viruses, with their sizes being proportional to mean relative abundances. The cutoff Spearman correlation coefficient and the adjusted *p*-value were |0.7| and 0.05, respectively. Degrees of distribution of virus-bacteria co-occurrence networks (**C**). Comparison of the alteration levels of the virus-bacteria co-occurrence networks based on the rates of removed modes (**D**). Degrees of viral nodes in virus-bacteria co-occurrence networks (**E**).

Virus-bacteria co-occurrence network analysis was carried out to determine the associations between viruses and bacterial organisms. We found that *Prevotella copri CAG:164* and *Prevotella copri* were more abundant in non-AF control patients, and multiple viruses, including *Hepacivirus G*, were negatively associated with this group (Figure 4A). We also observed that *Bacteroidetes*, including *Bacteroides vulgatus*, *Bacteroides eggerthii*, and *Bacteroides fragilis*, were positively correlated with *Synechococcus phage S-SM1* (enriched in control patients), suggesting *Synechococcus phage S-SM1* could infect these species (Figure 4B).

Structurally, the network of non-AF controls had 116 nodes, 183 edges, and 28 linkages, while that of AF cases encompassed 88 nodes, 117 edges, and 31 linkages (Supplementary Table 4). The densities of the networks in non-AF controls and AF cases were 0.0274 and 0.0306, respectively. The degrees of network distribution differed between the non-AF controls and AF cases (Figure 4C). Despite the higher number of linkages in the network of AF cases, its natural connectivity was reduced than that of the network generated in non-AF controls (Figure 4D). Next, entropies were assessed for these networks, and the control network (1.7138) showed an elevated value compared with that of AF cases (1.4171), indicating reduced randomness of potential virus-bacteria interactions, while the linkage of viruses to bacteria was increased. Specifically, virus-bacteria linkages increased from 28 in control patients to 31 in AF cases (Figure 4E), suggesting the linkage is pervasive across non-AF individuals to AF patients.

To characterize the relationship between gut bacteriome and virome, the association of the α diversity parameters of the bacteriome and the virome. In control patients, significant correlations were found between intra-kingdom and trans-kingdom α diversity parameters. However, associations of intra- and trans-kingdom α diversity parameters were attenuated in the AF group, being more severe from PAF to Pers AF<12 m and to Pers>12 m (Supplementary Figure 3A), indicating elevated dysbiosis of the gut microbiome in AF. Furthermore, the associations of bacterial species with viruses were examined in non-AF controls and AF patients. The altered virus-bacteria associations in AF were driven by both depleted and newly acquired ones; meanwhile, these correlations were lower in AF cases versus control patients (Supplementary Figure 3B). Notably, the associations of the viruses *Synechococcus phage S−SM1* and *Cronobacter phage CR5* with bacterial species were very tight in control individuals but markedly dampened in AF cases. The above findings suggested a changed viral-bacterial relationship in AF, with viruses and bacterial organisms becoming intertwined and more specialized, further revealing the significance of inter-kingdom equilibrium among gut viruses and bacterial organisms in human health.

### Association of the gut viral signals with the risk of AF recurrence

Given the significance of the risk of AF recurrence after radiofrequency catheter ablation, whether the gut virome could help predict recurrent AF (RAF) was examined to identify individuals who could highly benefit from catheter ablation. In this work, we constructed a gut virome-dependent signature for assessing the risk of AF recurrence.

**Figure 5:**
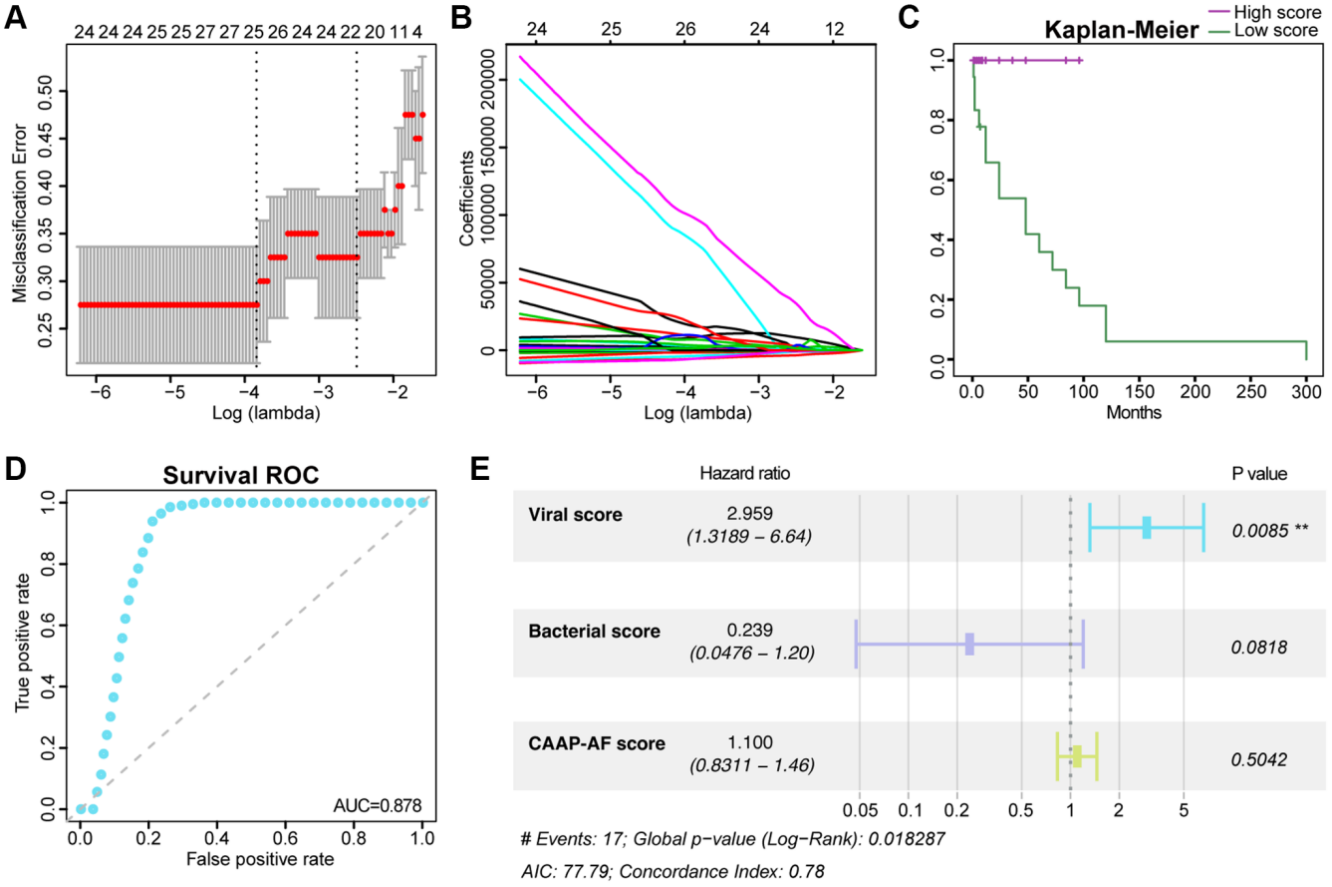
**Association of the gut viral signals with the risk of recurrent AF.** The tuning index (lambda) was identified utilizing the LASSO. Then, receiver operating characteristic (ROC) curve analysis was performed, and the AUC was plotted against log (lambda) (**A**). Dotted vertical lines represent the optimal values based on the minimum criteria and one standard error of the minimum criteria (1-SE criteria). Coefficients versus log (lambda) are shown (**B**). Kaplan-Meier curve analysis of overall survival based on the viral score (**C**). Survival ROC curves for the viral score (**D**). Forest plot of multivariate Cox regression analysis of virome-related risk groups and baseline patient covariates (**E**). Hazard ratios and 95% CIs are shown. Abbreviations: C-index: concordance index; CI: confidence interval.

First, taxa with the highest predictive values in AF recurrence were selected by LASSO analysis. Totally 21 viruses of all candidates (124 viruses with differences between the non-RAF and RAF groups with Wilcoxon rank-sum test *q* < 0.05) retained statistical significance, showing non-zero coefficients in 40 AF cases (Figure 5A, 5B). Then, a risk score (viral score based on a linear combination of the 21 viral taxa-based markers) was obtained. The value of −0.5727 was used as a cut-off for the viral score to determine the high- and low-risk groups of RAF cases by the nearest neighbor estimation (NNE) method. Kaplan-Meier curves showed that the high-risk group had an increased risk of RAF compared with low-risk individuals (*P* = 0.002, Figure 5C). The AUC of the time-dependent ROC curve for the viral score was 0.741 for sinus rhythm maintained on an average follow-up of 15.6 ± 12.57 months (Figure 5D). Finally, we found that the viral score (HR = 2.959, 95% CI: 1.3189−6.64, *p* = 0.0085) independently predicted prognosis upon adjustment for potential confounders, e.g., the bacterial score which was built in our previous study [15] (HR = 0.239, 95% CI: 0.0476−1.20, *p* = 0.0818) and a clinical scoring system called CAAP-AF score (HR = 1.100, 95% CI: 0.8311−1.46, *p* = 0.5042) (Figure 5E, Supplementary Table 5).

## DISCUSSION

Based on metagenomics data, the current work described the first and an in-depth human gut viral signature in AF patients. As demonstrated above, AF cases had an altered gut viral signal, characterized by increased viral diversity and changed viral composition. In addition, marked functional alterations in the gut virus were detected in AF, indicating essential roles for viruses in virus-host interactions. Furthermore, a virus-based strategy was utilized to build a predictive model for RAF after ablation. Moreover, we provide initial support regarding the virus as a vital part of the microbiome ecological network.

The human gut contains a vast array of viruses that are highly diverse and stable [16]. Disordered gut virome has been revealed in several human diseases [17], including colorectal cancer [18, 19], ulcerative colitis [20], nonalcoholic fatty liver disease [21], carcinogenesis [22], diabetes mellitus [23] and hypertension [24]. There are few studies examining the relationship between gut viruses and diseases, and the substantial role of gut viruses in human health is unknown. Viruses might regulate the compositions of the microbial community, therefore affecting the functions of the microbiota and disease progression [25]. Bacteriophages change bacterial community composition, promoting colonization by driver bacterial species. Then, passenger bacterial organisms initiate biofilm formation, which increases with phage-induced dispersal. Epithelial cell transformation, tight junction disruption, and bacterial infiltration also occur. Bacteria step into a dysbiotic microenvironment and may thrive off specific metabolites, which are disease-related and participates in the progression of the disease [26–28].

Viral richness across the human life stages was assessed in healthy, western individuals. Across the human lifespan in the human gut virome database, the highest overall viral richness was observed in infants and adults and there were significant increases between children and adults, and significant decreases between adults and elderly individuals [29]. Notably, the tendency of the viral diversity, which increases with aging and decreases between adults and elderly individuals, is similar to the age-dependent patterns revealed in western individuals. The role of aberrant gut viruses in the pathogenesis of age-related AF remains for further study.

Previous reports have suggested that viral markers may help diagnose pathologies and predict therapeutic responses [30, 31]. In the current study, multiple viruses had elevated levels and significant between-group variations, including *Synechococcus phage S-SM1*, *Cronobacter phage CR5*, and *Staphylococcus phage SPbeta-like*. The above viruses are firstly linked to AF in this report, and their functions in the gut microbiota remain largely undefined. The random forest model constructed based on viral species may provide some clues for the screening and detection of occult AF, which refers to AF with no typical clinical symptoms and no clear history detected by ECG monitoring. Occult AF accounts for 15%–40% of the total AF and is also a significant cause of cryptic stroke [32]. Therefore, it is of great clinical significance to carry out effective screening. The gut virome might be of specific value for the screening and stratification of the high-risk population for occult AF. In addition, altered gut virome independently predicted AF recurrence. Catheter ablation is broadly utilized in clinical AF, with high efficacy. However, high post-ablation recurrence demands the development of tools capable of improving the selection of individuals who would potentially benefit from ablation. Although no clear association of viruses with AF has been reported, we examined viruses as potential markers for detecting asymptomatic latent AF or building a predictive model for RAF.

Application of the gut virome mainly targets the modulation of the gut bacteriome, where lytic bacteriophages could reshape the structure of the gut microbiome [33]. With growing metagenomic evidence, the engineering of viral genomes could become a patient-specific therapy as a “final destination” [34]. For example, *Caudovirales* and *Microviridae* may be applied for future phage-specific therapies in Crohn's disease, and inhibiting bacteriophage infection of the respective bacterial hosts might also curve the natural course of inflammatory bowel diseases [35].

Finally, a large gap remains between human intestinal virome and AF. The current study is not typical virome research but a study mining viral signal from the vastly available bulk gut metagenomic sequencing data, which is accompanied by the limitation of omission about the viruses which need to be enriched during sampling purification. A standard virome study based on the deep shotgun metagenomic sequencing of virus-like particles-derived DNA should be performed to explore the role of the viral community in disease development. Further studies such as fecal virome transplantation trying to examine whether the AF phenotype is transferrable by altered gut virome and the subsequent effects on pathological changes related to atrial remodeling are still needed.

Overall, we described the altered gut viral profile in AF patients based on metagenomic data, which could be summarized as increased viral diversity, disturbed viral composition and functions, and disordered linkage between virus and bacteria. In addition, viruses were shown to have a superior predictive value for AF recurrence after ablation.

## MATERIALS AND METHODS

### Description of the study population of the metagenomic data

The whole-metagenome sequencing data analyzed in the current study were acquired from our previously published human non-valvular AF study (PRJEB28284) [6], which examined 50 non-AF control and 50 non-valvular AF cases in northern China. Fifty patients with nonvalvular AF were consecutively enrolled in the Heart center of Beijing Chaoyang Hospital since March 2016, and 50 individuals as matched controls were enrolled from the Kailuan cohort who received biennial medical examination in Kailuan General Hospital. Individuals with a history of heart failure, coronary heart disease, structural heart disease, comorbidities (inflammatory bowel diseases, irritable bowel syndrome, autoimmune diseases, liver diseases, renal diseases, or cancer), or use of antibiotics or probiotics in the past one month were excluded.

According to AF history and cardiogram manifestations [1], the 50 AF patients were assigned to the paroxysmal (PAF, *n* = 30) and persistent (psAF, *n* = 20) groups. Subsequently, the psAF cases were grouped into the <12-month (Pers<12 m, *n* = 12) and >12-month (Pers>12 m) groups, reflecting cases with psAF durations shorter and longer than one year, respectively. In addition, 40 AF patients underwent radiofrequency catheter ablation during the hospital stay. AF recurrence (RAF) occurred in 17 AF cases after a follow-up of 15.6 ± 12.57 months [36].

As previously reported [6–8, 36], AF patients were older, with more females than males, with a higher incidence of type 2 diabetes mellitus (T2DM), lower total cholesterol serum levels, and a higher incidence of medications compared to the non-AF CTR group. The baseline clinical characteristics among the different AF types were similar (PAF vs. psAF; Pers< 12 m vs. Pers> 12 m; non-RAF vs. RAF), with no remarkable difference in terms of age, sex, gender, body mass index, hypertension, T2DM, fasting blood glucose, serum creatinine, or alanine aminotransferase. The information about the treatment history of AF patients has been provided in Supplementary Table 6, including renin-angiotensin system inhibitors, amiodarone, statin, metformin, and radiofrequency ablation history. The study had approval from the ethics committees of Beijing Chaoyang and Kailuan General Hospitals. Each patient provided signed informed consent at the time of enrolment.

### Assembly of metagenomic data, taxonomic assignment, and abundance profiling

MEGAHIT v1.1.3 was applied to assemble using presets meta-large, with a k-mer ranging from 27 to 127, step 10. Only contigs >1,000 bp were retained for subsequent assessment for high predictive accuracy for viral sequences [37]. Viral sequences from these contigs were predicted and annotated based on the Kraken virus database (default parameters) and the taxonomies of various viruses [38]. The viral sequences were utilized for constructing a viral database to calculate viral composition in 100 fecal specimens. Contig coverage (Reads Per Kilobase per Million mapped reads, RPKMs) was determined with Bowtie2 (default parameters), normalizing to contig length and the number of mapped reads in a given specimen [39]. Average RPKM for a given virus was determined in every specimen for relative abundance calculation.

### Rarefaction curve and diversity analyses

Rarefaction analysis based on relative abundance levels of viral contigs and viruses was conducted to evaluate whether the sequencing data were sufficiently deep and the current sample size was optimal for the present analysis. The patient population underwent 100-time random sampling with replacement, and common viral contigs/viruses and viral contigs/viruses joint in sample pairs were assessed, and R v2.15.3 (vegan package) was utilized for plotting. To determine taxonomic diversity, the total number of viruses, α (within-individual) diversity indexes such as Simpson index, Shannon index, Pielou index, and Chao 1 richness, and β (between-individual) diversity using nonmetric dimensional scaling (NMDS) according to Bray-Curtis differences at the species level were calculated.

### Virome function analysis

Virome functions were classified by annotating all viral-contig derived reads with HUMANN2 v0.9.4 (default parameters). Gene ontology (GO) terms, Pfam protein family identities, and KEGG orthology data were utilized to predict functions, and abundance levels were determined in RPK (reads per kilobase) [20].

### Virus and bacteria co-occurrence network analysis

To determine the associations of bacterial and viral communities in non-AF controls and AF patients, the top 100 viruses and top 100 bacterial species based on their relative abundances were selected, and virus-bacteria linkages were further examined. Spearman correlation analysis was carried out to determine these associations, using the “cor” function in R and correcting for multiple testing by the Benjamini-Hochberg method. The cutoffs for the correlation coefficient and adjusted *p*-value were 0.7 and 0.05, respectively. The igraph package (v1.1.2) in R was used to quantitate distribution, node betweenness, and the network’s natural connectivity. Entropy for every network was obtained based on node degree using the entropy package (v1.2.1) in R. Finally, associations with statistical significance were imported into Cytoscape for visualizing virus-bacteria bipartite networks, in which nodes and edges represent viral and bacterial entities, and positive and negative associations of bacteria with viruses, respectively [24].

### Design and validation of a predictive model for RAF

The least absolute shrinkage and selection operator (LASSO) algorithm was utilized to select indexes with the highest predictive efficiencies based on significant taxa between the non-RAF and RAF groups. Viral scores were obtained for all cases by a linear combination of the retained taxa weighted by their respective coefficients. Internal validation was carried out based on a method reported in our previous study [36].

### Data availability

Raw data was available at European Nucleotide Archive via PRJEB28384; the datasets generated and analyzed in this study are available from the corresponding author upon reasonable request.

## Supplementary Materials

Supplementary Figures

Supplementary Tables 1 and 5

Supplementary Tables 2-4 and 6
