# Supplementary material for: Alterations of gut viral signals in atrial fibrillation: complex linkage with gut bacteriome
Source: Aging (Albany NY). 2022 Aug 18;14(16):6537–53. doi: 10.18632/aging.204222 (PMC9467389; doi:10.18632/aging.204222)
Supplement: Supplementary Figures [file aging-14-204222-s001.pdf]

SUPPLEMENTARY FIGURES

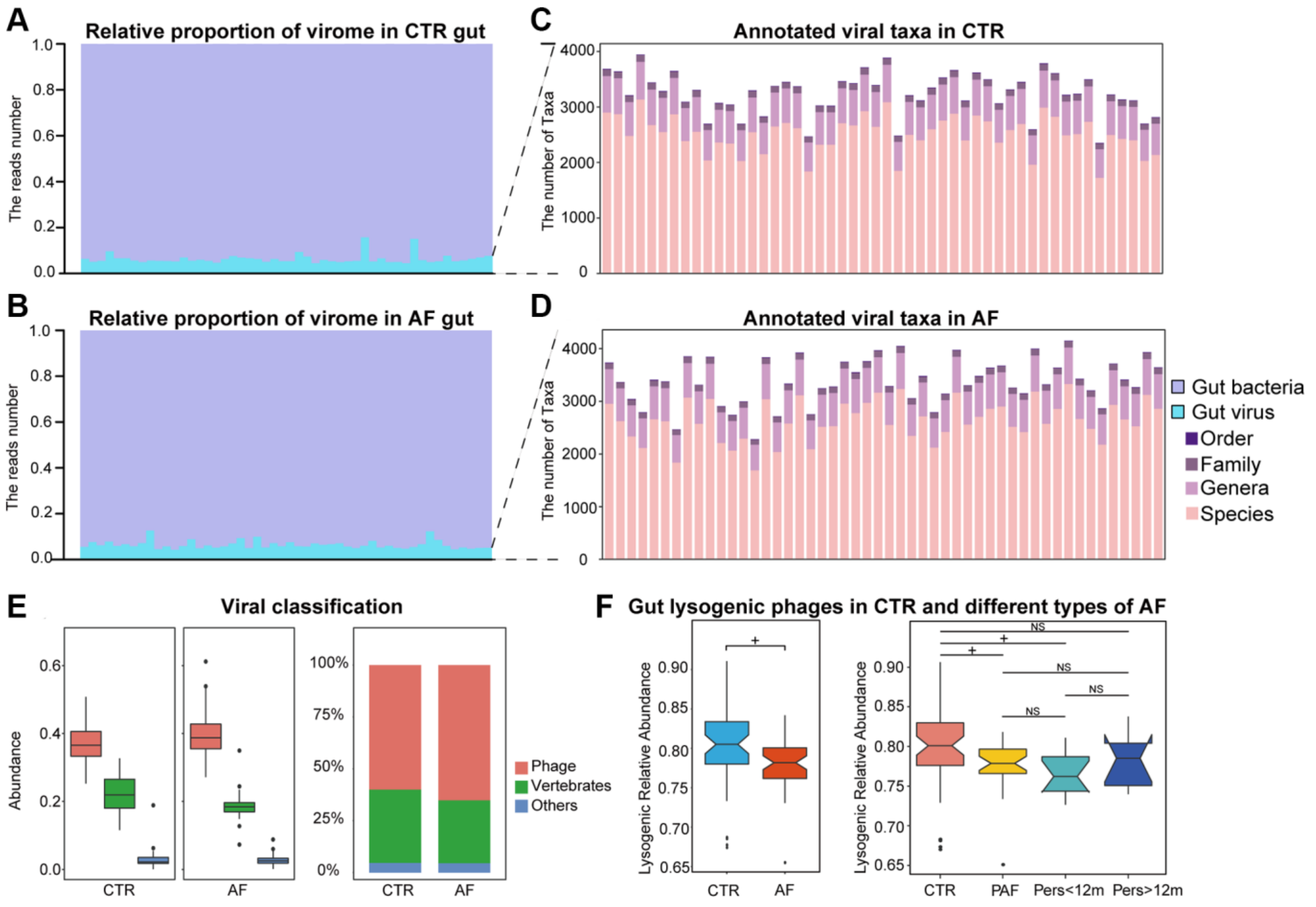

Supplementary Figure 1. Taxonomic profile of the gut viral signals in AF. Relative proportions of bacterial and viral read numbers in the gut of controls (**A**) and AF patients (**B**). Taxonomic landscapes of the numbers of viral taxa at the order, family, genera, and species levels in controls (**C**) and AF patients (**D**). The box plot shows the abundance of vertebrate-infecting viruses and phages (**E**) and lysogenic phages (**F**). Boxes are interquartile ranges; lines denote medians, and circles are outliers. *indicates $p < 0.05$, and NS indicates no significant difference based on the *t*-test.

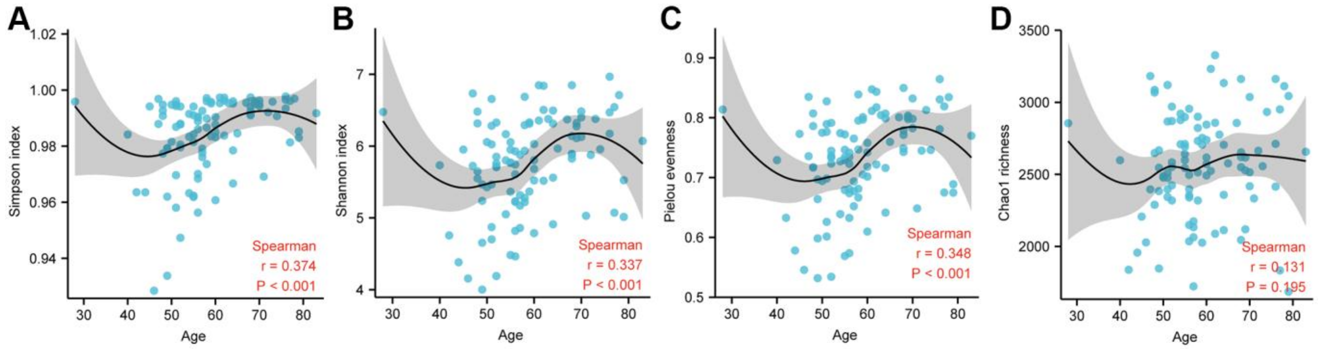

Supplementary Figure 2. Spearman correlation between age and viral diversity including (**A**) Simpson index, (**B**) Shannon index, (**C**) Pielou evenness, and (**D**) Chao1 richness.

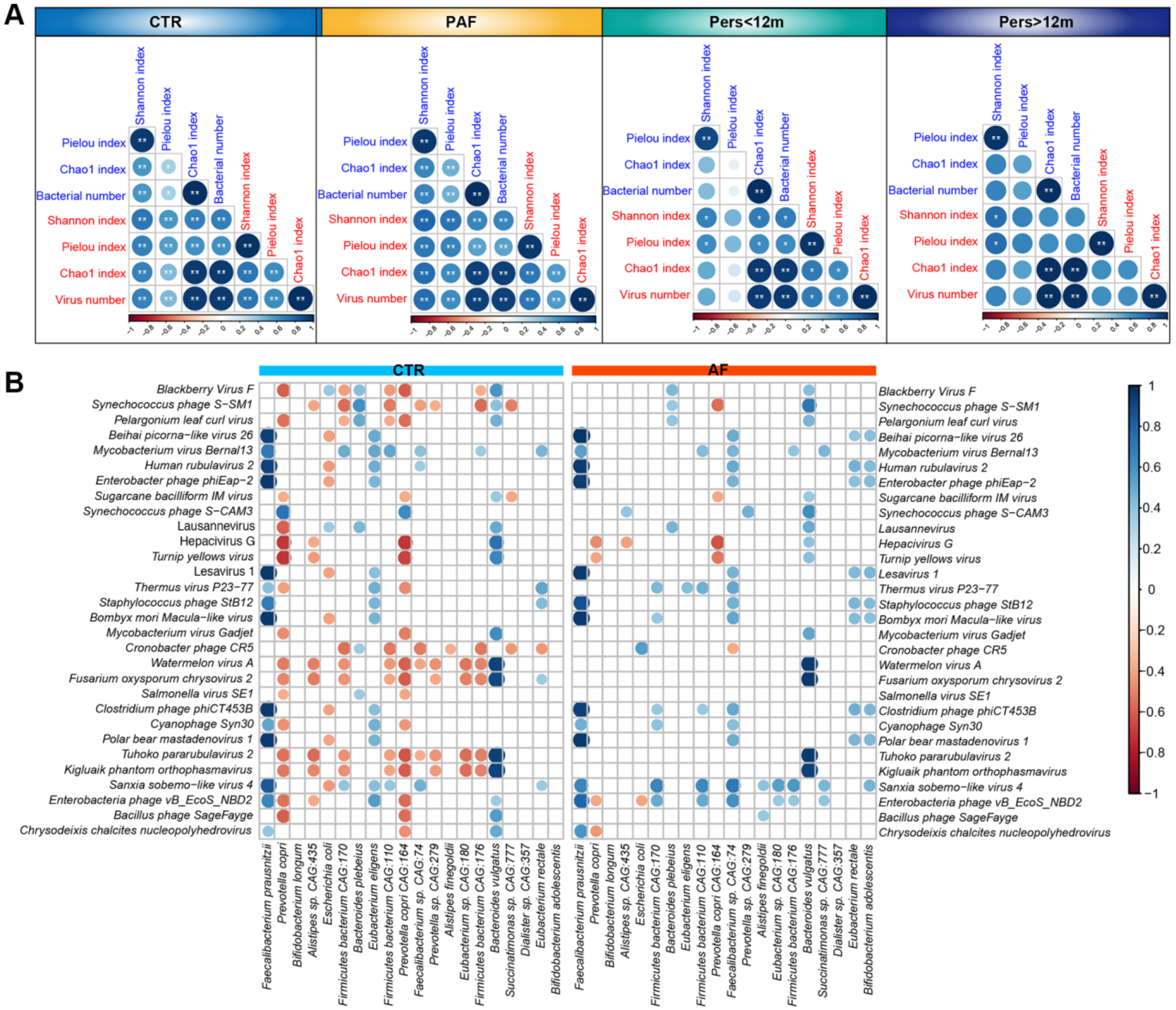

Supplementary Figure 3. Trans-kingdom linkage between gut virus and bacteria in AF. (**A**) Correlations between the α diversity indexes of intestinal bacteria and viruses in non-AF individuals, PAF, Pers<12 m and Pers>12 m. Spearman's correlation coefficients were determined for all pairs. *$P < 0.05$ and **$P < 0.01$ indicate statistical significance. The circle's size and intensity reflect the correlation's magnitude, the darker and greater the circle, the tighter the correlation. (**B**) Associations of the most common 30 viruses with the most common 20 bacterial species in non-AF controls and AF patients. Spearman's correlation coefficients were determined for all pairs. Only correlations with statistical significance are shown. Blue and orange circles denote positive and inverse correlations, respectively. The circle's size and intensity reflect the correlation's magnitude, the darker and greater the circle, the tighter the correlation.
