# Supplementary material for: Alterations of gut viral signals in atrial fibrillation: complex linkage with gut bacteriome
Source: Aging (Albany NY). 2022 Aug 18;14(16):6537–53. doi: 10.18632/aging.204222 (PMC9467389; doi:10.18632/aging.204222)
Supplement: Supplementary Tables 1 and 5 [file aging-14-204222-s002.pdf]

SUPPLEMENTARY TABLES

Supplementary Table 1. The association between potential confounders and viral diversity.

	**Spearman correlation analyses**							
	**Shannon index**		**Pielou evenness**		**Chao1 richness**		**Simpson index**	
	**Correlation**	***p* value**	**Correlation**	***p* value**	**Correlation**	***p* value**	**Correlation**	***p* value**
AF	0.499	<0.001	0.522	<0.001	0.168	0.095	0.567	<0.001
Age	0.337	0.001	0.348	<0.001	0.131	0.195	0.374	<0.001
Gender	0.004	0.972	0.036	0.726	−0.153	0.130	0.040	0.691
BMI	0.048	0.638	0.032	0.752	0.053	0.605	0.032	0.751
TC	−0.215	0.032	−0.229	0.022	−0.107	0.289	−0.248	0.013
T2DM	0.035	0.728	0.058	0.569	−0.102	0.311	0.091	0.370
HTN	0.018	0.858	0.024	0.810	−0.041	0.683	0.006	0.956
Statin	−0.034	0.740	−0.035	0.727	−0.042	0.675	0.018	0.861
Metformin	0.089	0.379	0.124	0.219	−0.086	0.395	0.140	0.165
RASI	0.312	0.002	0.304	0.002	0.145	0.150	0.296	0.003
Amiodarone	0.382	<0.001	0.359	<0.001	0.379	<0.001	0.358	<0.001
	**Multivariate Linear Regression**							
	**Shannon index**		**Chao richness**		**Pielou evenness**		**Simpson**	
	Beta	***p* value**	***Beta***	***p* value**	***Beta***	***p* value**	***Beta***	***p* value**
AF	0.386	0.003	0.064	0.631	0.419	0.001	0.380	0.004
Age	0.111	0.324	0.112	0.353	0.108	0.339	0.175	0.131
Gender	−0.092	0.333	−0.171	0.094	−0.074	0.432	−0.088	0.363
BMI	−0.051	0.587	0.053	0.600	-0.067	0.476	−0.090	0.351
TC	−0.064	0.532	−0.126	0.252	−0.049	0.632	0.009	0.931
T2DM	−0.170	0.192	−0.100	0.473	−0.171	0.187	−0.095	0.473
HTN	−0.078	0.443	−0.168	0.124	−0.058	0.566	−0.185	0.078
RASI	0.125	0.267	0.007	0.951	0.136	0.223	0.133	0.246
Statin	−0.149	0.121	−0.114	0.266	−0.147	0.126	−0.067	0.492
Amiodarone	0.199	0.050	0.377	0.001	0.153	0.127	0.091	0.378
Metformin	0.103	0.423	−0.068	0.621	0.127	0.321	0.095	0.469

Abbreviations: AF: atrial fibrillation; BMI: body mass index; HTN: hypertension; DM: diabetes mellitus; TC: total cholesterol; HTN: hypertension; RASI: renin-angiotensin system inhibitor.

Supplementary Table 5. Viral, bacterial, and CAAP-AF score for each sample.

ID	Viral score	Bacteria score	CAAP-AF score
no_reAF1	−1.643436274	−0.438057175	8
no_reAF2	−0.572739537	−0.559589758	4
no_reAF3	−1.112909752	−0.41292474	5
no_reAF4	−1.720926254	−0.480547348	4
no_reAF5	−1.663225355	−0.629424774	3
no_reAF6	−1.207326045	−0.457982056	2
no_reAF7	−0.794490746	−0.403373535	7
no_reAF8	−1.449086837	−0.924431659	4
no_reAF9	−0.690488681	−0.372348362	3
no_reAF10	−1.590535439	−0.83456788	5
no_reAF11	−1.454642971	−1.018637523	1
no_reAF12	−1.38143236	−0.522411896	3
no_reAF13	−1.17920171	−0.784698581	6
no_reAF14	−0.745314246	−0.941740217	7
no_reAF15	−1.446141423	−0.846711054	3
no_reAF16	−2.376555056	−0.406248875	4
no_reAF17	−1.028053231	−0.121126375	0
no_reAF18	−0.853312857	−1.094805179	2
no_reAF19	−1.086303952	−0.405425016	1
no_reAF20	−1.390471095	−0.474613524	1
no_reAF21	−1.001423967	−0.838099047	4
no_reAF22	−1.534434775	−0.913203781	3
no_reAF23	−0.39917529	−0.515052	1
reAF1	0.857412367	0.130452354	5
reAF2	0.662074237	−0.114841987	2
reAF3	0.788297736	−0.430826956	4
reAF4	0.808764009	−0.072169315	1
reAF5	0.089692379	0.094527915	4
reAF6	1.711957888	0.744633894	8
reAF7	1.333083101	−0.166743886	7
reAF8	0.539843254	−0.285436126	7
reAF9	0.67832355	0.1271324	2
reAF10	1.638005186	0.942875207	4
reAF11	1.182311213	−0.386605779	4
reAF12	0.194674374	−0.160757943	4

reAF13	1.581301599	−0.215528759	6
reAF14	0.785256346	0.821312269	5
reAF15	0.2598357	0.680733825	6
reAF16	0.638192391	−0.413989751	7
reAF17	0.067054588	0.611781801	8

Abbreviations: viral score = (−0.4191 *(Intercept)) + (−151.9898 *Lepidopteran hudovirus) + (−824.8187 *Acanthocystis turfacea chlorella virus 1) + (−32.1303 *Synechococcus phage S-RIM8) + (−36.6109660550033 *Agrotis segetum nucleopolyhedrovirus B) + (−1001.4687 *Mokola lyssavirus) + (1046.1101 *Rhodococcus phage REQ1) + (564.2624 *Chicken anemia virus) + (1061.2306 *Escherichia virus HX01) + (1216.3961 *Guajara orthobunyavirus) + (−879.1352 *Escherichia virus ADB2) + (−80.5973 *Azospirillum phage Cd) + (−3751.0734 *Helicoverpa armigera granulovirus) + (1722.6372 *Salmonella phage SPN3UB) + (73.0350 *Listeria virus P100) + (112.2706 *Beihai zhaovirus-like virus 1) + (9441.7402 *Maize rayado fino virus) + (34369.6655 *Ribgrass mosaic virus) + (568.4422 *Wuhan millipede virus 3) + (2657.2776 *Wuhan insect virus 14) + (3857.4019 *Narcissus mosaic virus) + (1303.9541 *Paracoccus phage vB_PmaS-R3).
